# Live-attenuated foot-and-mouth disease virus vaccine engineered by codon deoptimization induces a strong protective immune response in cattle

**DOI:** 10.1038/s41541-025-01368-7

**Published:** 2026-01-24

**Authors:** Sarah E. Attreed, Christina Silva, Monica Rodriguez-Calzada, Ryan D. Heimroth, Michael Oldakowski, Carolina Stenfeldt, Paul Azzinaro, Edward Spinard, Aishwarya Mogulothu, Steffen Mueller, Bruce Taillon, Jonathan Arzt, Elizabeth Rieder, Teresa de los Santos, Fayna Díaz-San Segundo, Gisselle N. Medina

**Affiliations:** 1 Plum Island Animal Disease Center, U.S. Department of Agriculture, Agricultural Research Service, Greenport, NY USA; 2 National Bio- and Agro-Defense Facility, U.S. Department of Agriculture, Agricultural Research Service, Manhattan, KS USA; 3 PIADC Research Participation Program, Oak Ridge Institute for Science and Education (ORISE), Oak Ridge, TN USA; 4 Department of Diagnostic Medicine and Pathobiology, College of Veterinary Medicine, Kansas State University, Manhattan, KS USA; 5 Department of Pathobiology and Veterinary Science, University of Connecticut, Mansfield, CT USA; 6 Codagenix, Farmingdale, NY USA; 7 Present Address: U.S. Department of Agriculture, Animal and Plant Health Inspection Service, National Veterinary Services Laboratories, Ames, IA USA; 8 Present Address: Office of Biodefense, Research, Resources and Translational Research, Division of Microbiology and Infectious Diseases, National Institute of Allergy and Infectious Diseases, Bethesda, MD USA

**Keywords:** Diseases, Immunology, Microbiology

## Abstract

Foot-and-mouth disease (FMD) is a fast-spreading, economically devastating veterinary viral disease. While inactivated vaccines have contributed to FMD control worldwide, recent outbreaks in Europe and Asia highlight the need for new control strategies. Live-attenuated virus (LAV) vaccines provide strong and long-lasting protection. We previously demonstrated that deoptimization of various viral coding regions results in attenuated foot-and-mouth disease virus (FMDV). Here, an FMDV A24Cruzeiro LAV with codon deoptimized P2/P3 regions (A24-P2/P3Deopt) and markers differentiating infected from vaccinated animals (DIVA) was tested for safety/efficacy in cattle. Animals inoculated intradermolingually (IDL) with 10^6^ or 10^7^ pfu of A24-P2/P3Deopt for safety testing exhibited no clinical signs, viremia or viral shedding for 28 days post inoculation (DPI). To assess efficacy, cattle were subcutaneously inoculated either once with 10^5^ pfu or twice (0- and 14-dpi) with 10^4^, 10^5^ or 10^6^ pfu A24-P2/P3Deopt. No animal developed signs of disease post-inoculation. All prime-boost vaccinated animals developed strong neutralizing antibody responses that were protective against challenge with WT FMDV A24. Moreover, vaccinated sera showed strong cross-reactivity against several A strains and serotype Asia1. Our work demonstrates that codon deoptimization is a viable technology to derive novel LAV candidates that are safe, immunogenic and efficacious against FMD in cattle.

## Introduction

Foot-and-mouth disease (FMD) is a highly transmissible disease of even-toed ungulates, characterized by the development of vesicles on the mouths, feet and teats of affected individuals^1^. FMD is known to affect over 70 wild and domestic species such as swine, cattle, sheep, goats and cervids^1^. Given the host range of the disease, it is of great agricultural interest around the world, particularly in pig and ruminant agriculture. FMD is one of 26 multi-species reportable terrestrial diseases for the World Organization for Animal Health (WOAH)^2^. While mortality from FMD is low, the disease is of great economic importance as it is highly transmissible and there are restrictions on the movement and export of animal products from FMD-endemic countries. Therefore, maintenance of FMD-free status is a valuable economic asset for all countries worldwide.

Foot-and-mouth disease virus (FMDV) is the causative agent of FMD and is a member of the *Picornaviridae* family along with enteroviruses such as Poliovirus. FMDV is a single-strand, positive sense RNA virus of ~8.5 kb composed of a single open reading frame (ORF), flanked by 5’ and 3’ untranslated regions (UTRs). The ORF is divided into four main regions: Leader proteinase (L^pro^), P1, P2 and P3. Upon translation, the immature polyprotein is processed by 2A (a non-canonical protease that cleaves immature proteins by translational ribosome skipping) as well as the classical viral proteinases L^pro^ and 3C^pro^ into structural proteins—1A (VP4), 1B (VP2), 1C (VP3), and 1D (VP1)—as well as nonstructural (NS) proteins—2A, 2B, 2C, 3A, three distinct 3B genome-linked proteins (Vpg)^3^, 3C^pro^ and RNA-dependent RNA polymerase 3D^pol^.

Current control measures in FMD-free countries include culling of infected and contact animals, movement restrictions of infected and contact animals and sometimes prophylactic ring vaccination with an adjuvanted inactivated vaccine^4^, which, when combined with Montanide ESSAI IMS D 12802 VG PR aqueous adjuvant, can confer protective immunity within 4 days^5^. Another candidate vaccine under conditional license in the US employs a replication-defective adenovirus 5 (Ad5) encoding only the P1 capsid, 2B and 3C protease domains^6^. While the Ad5-FMD vaccine has been shown to confer immunity in approximately 7 days, similar to the commercially available inactivated vaccine, neither confers long-lasting immunity, which is often a function of strong T cell engagement during the post-vaccination period. To address the desire for more durable immunity as well as to possibly broaden the cross-neutralization potential of the vaccines available for FMD, other methods of vaccine preparation are being employed. One of the most successful methods for vaccination against viral infections involves the use of live attenuated virus (LAV) vaccines, which can replicate in the host, eliciting a more robust B and T cell response. Some of the most successful vaccination campaigns against viral pathogens have utilized LAVs, including smallpox^7^ and Rinderpest^8^, which were both eradicated, and the oral polio vaccine that is still part of the eradication strategy^9,10^. However, LAVs may be prone to recombination and random mutation events that can result in reversion to virulence, increasing the awareness about vaccine safety. One method that has been devised to simultaneously reduce viral virulence as well as the risk of replication-driven recombination is codon deoptimization of the viral genome. Codon deoptimization (CD) and codon pair deoptimization (CPD) are strategies for virus attenuation built upon the concept that, while multiple codons exist for many common amino acids, these codons and codon pairs are not evenly employed. Rather, genomes show bias in the employment of some codons over the use of others in a species-specific manner. In CD and CPD, selected genomic regions can be rewritten in such a way as to swap out “preferred” codons or codon pairs for those less commonly encountered in the given genome. While the precise mechanisms of attenuation are unknown, it has been shown that synonymous codon substitutions can alter the co-translational protein folding mechanisms, which can increase degradation of these proteins in the cell^11^.

To date, CD/CPD have been utilized in the development of a number of LAV vaccine candidates. Vaccinia virus (VV), a DNA poxvirus—which serves as the backbone to many poxvirus vaccines—was found to be strongly attenuated in vivo by CD and to confer strong immunity to an otherwise fatal challenge of VV^12^. When applied to Zika virus, a positive sense ssRNA virus of the *Flaviviridae* family, CPD viruses exhibited reduced virus replication fitness and some induced sterilizing immunity in a mouse model, protecting against lethal challenge as well as vertical transmission during pregnancy^13^. An engineered deoptimized respiratory syncytial virus (RSV), a negative sense ssRNA virus of the *Pneumoviridae* family, resulted in minimal viral shedding when administered to African green monkeys that then produced a strong humoral and cellular immune response^14^. A construct with CPD targeted only to the surface glycoprotein G and F ORFs showed reduced viral replication fitness and temperature sensitivity. However, some viral fitness was regained over several passages, along with defective helper particles^15^. A 2021 paper by Chen et al. also found that a CPD RSV virus regained replication fitness under many serial passages, particularly through mutations in the phosphoprotein gene^16^. A more recent RSV vaccine candidate with CPD applied to seven of the virus’ eleven ORFs demonstrated retained temperature sensitivity over many passages and strong humoral immunogenicity in hamsters and protective efficacy as measured at three days post-challenge (dpc)^17^.

CPD has also been utilized in other picornaviruses, such as poliovirus. In one such study, where the capsid region was CPD, highly modified viral clones exhibited lower RNA yield and specific infectivity^18^. Furthermore, the authors found that reduced fitness was well-maintained over up to 25 serial passages, or about 50 viral replication cycles. In a separate study, Mueller et al. found that, for equal doses of viral particles, pathogenicity to the brain was significantly decreased in CPD infectious clones with the most replication deficient phenotype^19^. In a study of Enterovirus A71, a picornavirus that causes hand-foot-and-mouth disease, CD of the P1 region, along with a high-fidelity 3D polymerase, resulted in a genetically stable viral clone with decreased virulence and maintained antigenicity^20^. Finally, work from our own lab has consistently revealed slower growth kinetics and reduced virulence in CPD and CD FMDV across several serotypes and strains. When CPD was applied to the capsid (P1) region of FMDV A12 and the resulting attenuated strain tested in vivo, mice were able to tolerate inoculation doses up to 10,000 times higher than the WT dose needed to cause disease, yielding a safety margin of at least 4 logs, while the safety margin of A12 WT virus was only a single log^21^. In a study by Medina et al., where CPD was applied to the P1 region of A24 and Asia1, attenuated virulence and growth kinetics were observed in a serotype-independent manner both in mice and swine, though protection against later parental challenge was not observed in swine^22^. In another study, various strains were derived where CD was applied to one or both of the P2 and/or P3 regions of the FMDV A24Cruzeiro genome, then tested for safety and efficacy in mice and swine^23^. The P2-deopt and P2/P3-deopt strains were found to have reduced virulence in mice and elicited strong neutralizing antibody titers in both mice and swine. While the A24-P2/P3Deopt strain was also found to have reduced virulence in swine, as was found with the CPD A12-P1Deopt, A24-P1Deopt and Asia1-P1Deopt strains^22^, inoculation was found not to confer protection against later homologous challenge.

In the current study, we evaluated the FMDV A24Cruzeiro with P2/P3 CD region strain (heretofore A24-P2/P3Deopt) for both safety and efficacy as a LAV vaccine candidate in dairy cattle according to WOAH requirements. We demonstrate that the margin of safety in cattle for this strain is at least 3 logs, with a prime-boost schedule of 10^4^ pfu/animal conferring protection against later homologous challenge, while an intradermolingual (IDL) dose of up to 10^7^ pfu (the highest dose tested) resulted in neither clinical disease nor viremia or shedding in safety-tested cattle. Additionally, sera from inoculated cattle displayed broadly neutralizing capacity against a range of A strains and even the Asia1 serotype, suggesting the potential of such a vaccine during an outbreak scenario.

## Results

### Safety study

**Fig. 1:**
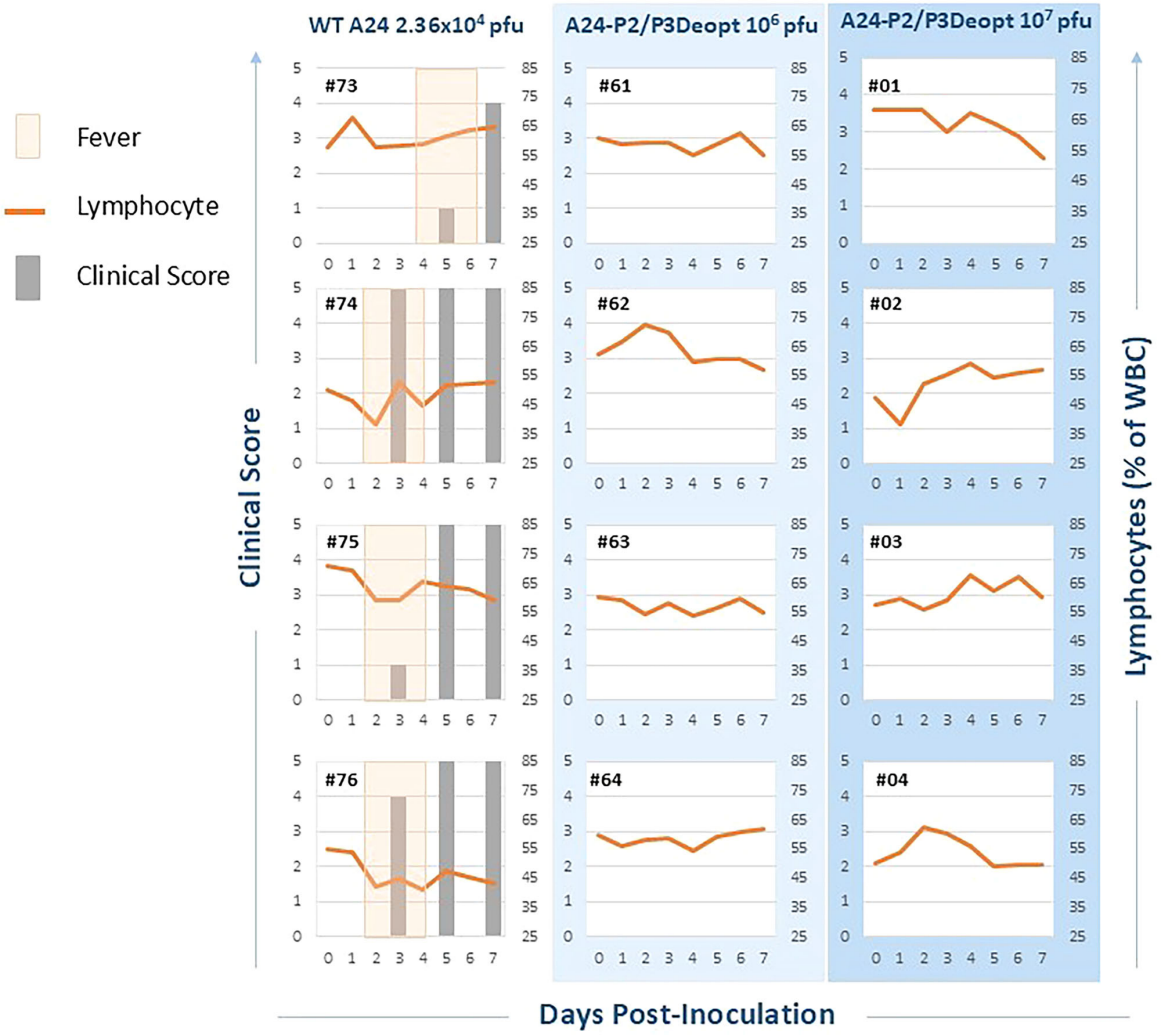
Clinical safety evaluation of FMDV A24-P2/P3Deopt. Six-month-old Holstein heifers of approximately 450 lb were intradermolingually inoculated with a FMDV A24 P2/P3 codon deoptimized live attenuated virus (10^6^ or 10^7^ pfu) or FMDV A24 wild type (2.36 × 10^4^ pfu). Cattle were assessed for clinical score (grey bars) on days 3, 5 and 7 post-inoculation. EDTA-treated blood was assessed for signs of lymphopenia daily (orange line) and rectal body temperature was assessed daily (periods of fever shown in tan shaded boxes). *n* = 4 cattle/time point/treatment group.

We decided to pursue use of A24-P2/P3Deopt as a LAV in cattle after observing significant attenuation during prior in vivo routine titration according to WOAH standards. To first test the safety of this candidate LAV, we inoculated cattle with A24-P2/P3Deopt intradermolingually (IDL). The WOAH requires that for inactivated virus vaccine potency assays, all control animals must develop typical FMD lesions in 3 out of 4 feet within 8 days of inoculation with 10,000 median bovine infectious dose (BID_50_) of WT FMDV^24^, equivalent to 2.36 × 10^4^ pfu of our WT A24 strain. In the present study, none of the animals IDL inoculated with either 10^6^ or 10^7^ pfu of A24-P2/P3Deopt virus developed vesicular FMD lesions or fever (Fig. 1). Mild, transient lymphopenia—a characteristic feature of FMD^25^—was observed in three out of four control (wild-type inoculated) cattle at 2 dpi (#74, 75 and 76) and in one out of four cattle in the 10^7^ pfu dose group at 1 dpi (#02).

**Fig. 2:**
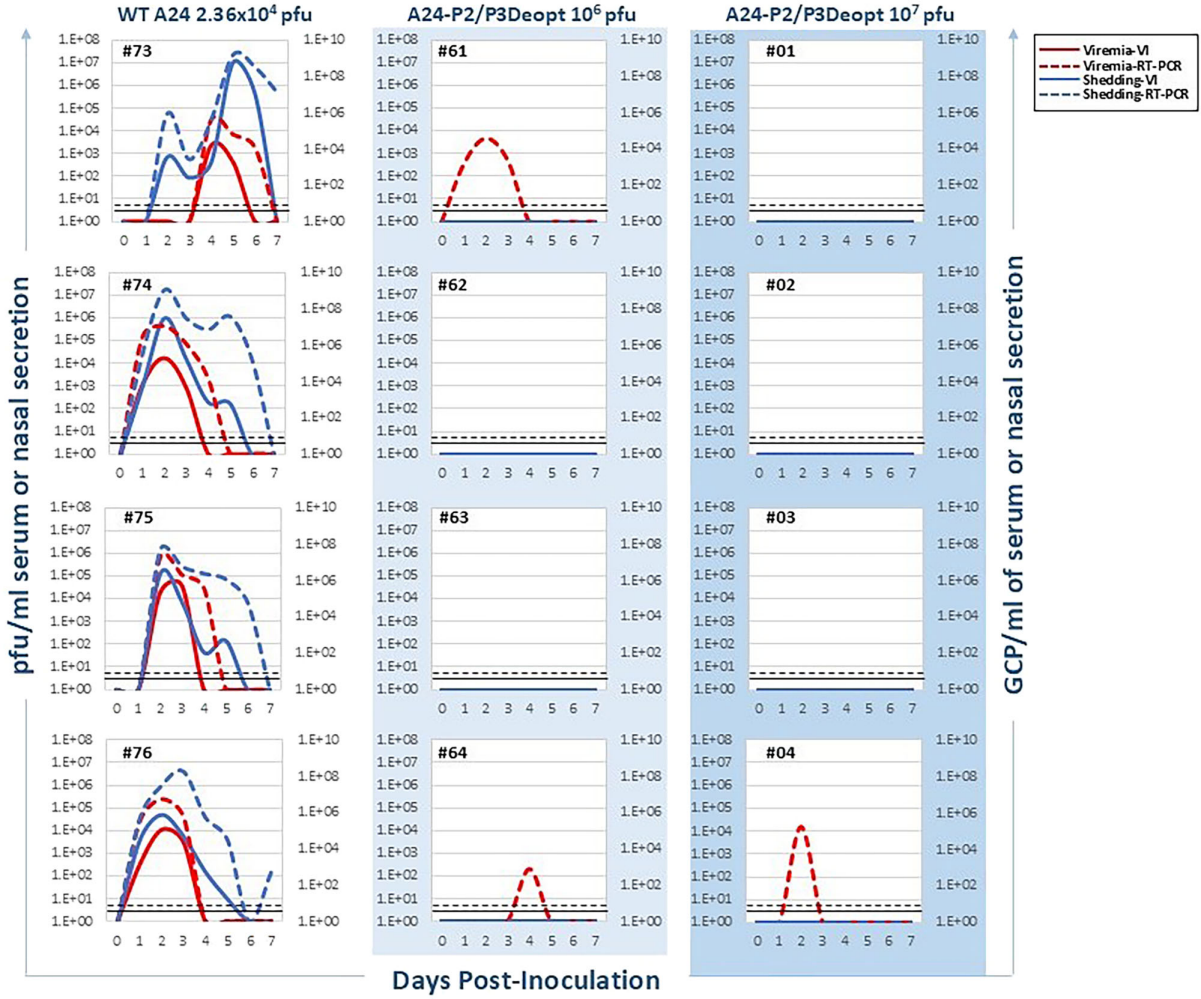
Viremia and virus shedding in A24-P2/P3Deopt in vivo safety study. Six-month-old Holstein heifers of approximately 450 lb were intradermolingually inoculated with a FMDV A24-P2/P3 codon deoptimized live attenuated virus (10^6^ or 10^7^ pfu) or FMDV A24 wild type (2.36 × 10^4^ pfu). Serum and nasal secretion samples were collected daily for a week to assess viremia and virus shedding, respectively. Viremia analyzed by virus isolation is expressed as pfu/ml (solid red line). Viremia analyzed by real-time RT-qPCR is expressed as genome copy particles/ml of serum (GCP/ml) (dashed red line). Virus in nasal secretion analyzed by virus isolation is expressed as pfu/ml (solid blue line), while that analyzed by real-time RT-qPCR is expressed as GCP/ml (dashed blue line). Lower limits of detection are represented by a black dashed line for RT-qPCR and a solid black line for virus isolation. *n* = 4 cattle/time point/treatment group.

During FMD progression in cattle, FMDV is typically shed in abundance as early as one day after infection with WT virus, which can lead to the rapid direct contact spread of the virus in both wild and domesticated herds of susceptible animals. In our study, following inoculation with FMDV A24 WT, we studied viral shedding (Fig. 2) by analyzing viral burden of nasal swabs by virus isolation, observing a peak at 10^4^ to 10^7^ pfu/ml and by RT-qPCR up to 10^8^ to 10^9^ genome copy particles (GCP)/ml. By contrast, no viral shedding was detected via either method following inoculation with either IDL dose of FMDV A24-P2/P3Deopt. When we assessed viremia by virus isolation in A24-WT inoculated animals, we found peak titers of about 10^3^ to 10^4^ pfu/ml and 10^5^ to 10^7^ GCP/ml when assessed by RT-qPCR. By contrast, we were unable to isolate infectious virus from the sera of cattle inoculated with either dose of FMDV A24-P2/P3Deopt virus. Rather, we observed only “RNemia” ranging between 10^2^ and 10^5^ GCP/ml in 3 out of 8 A24-P2/P3Deopt-inoculated animals (#61, 64 and 04), while RNemia was below the lower limit of detection for the remaining 5/8 FMDV A24-P2/P3Deopt inoculated animals.

**Fig. 3:**
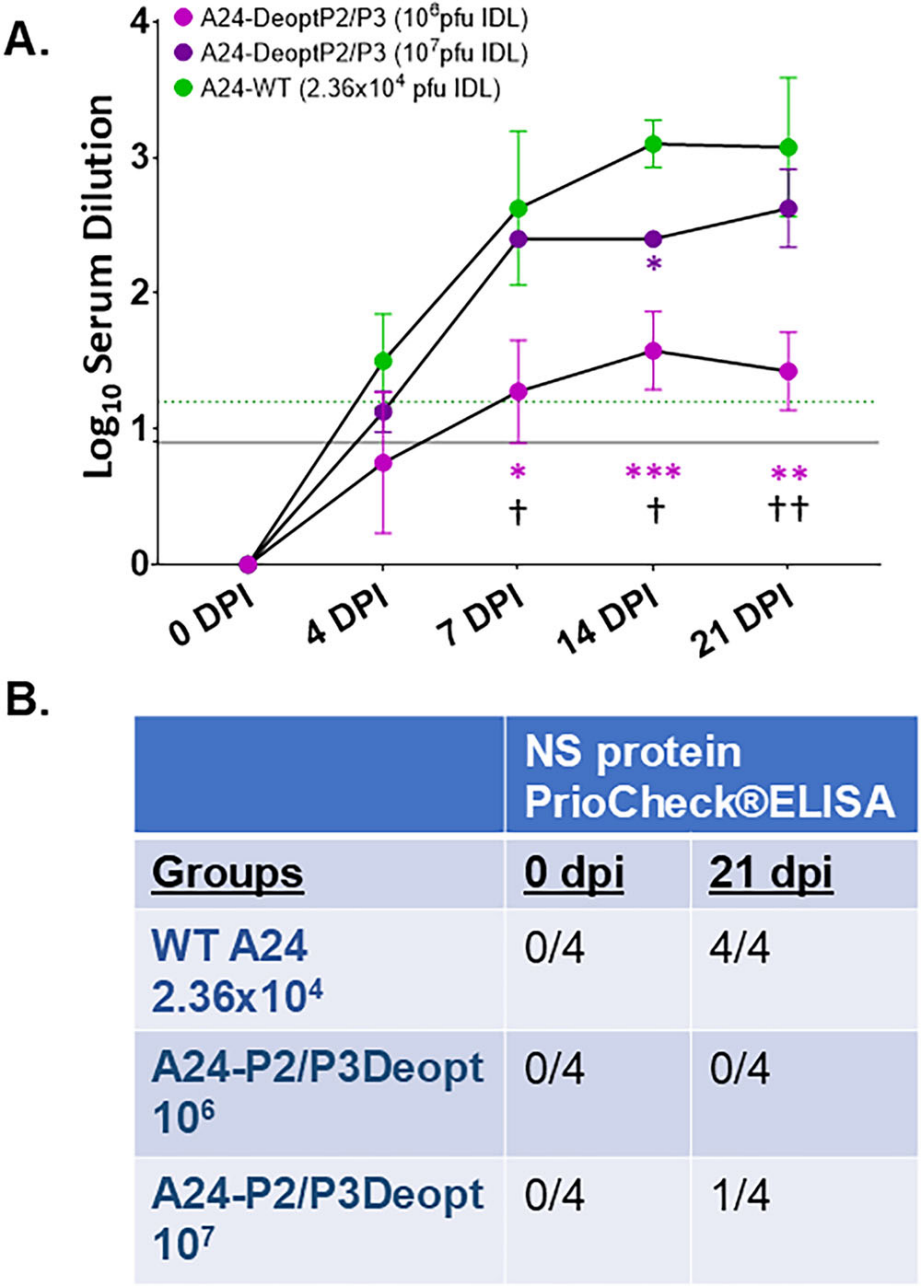
A24-P2/P3Deopt induces strong neutralizing antibody response in cattle. Six-month-old Holstein heifers of approximately 450 lb were intradermolingually inoculated with a FMDV A24-P2/P3 codon deoptimized live attenuated virus (10^6^ or 10^7^ pfu) or FMDV A24 wild type (2.36 x 10^4^ pfu). **A** At indicated time-points serum samples were collected to test for neutralizing antibodies (lower limit of detection represented by solid black line, while the protective titer is represented by a gray dashed line) or **B** antibodies against FMDV non-structural proteins (PrioCheck®ELISA). Results expressed as number of positive samples out of the total number of animals per group. *n* = 4 cattle/time point/treatment group. Neutralizing antibody titers assessed by a mixed-effects two-way ANOVA by treatment group and time point, followed by pairwise comparisons at each time point by treatment group with Tukey’s multiple comparison correction. Treatment group (*P* < 0.0001), time point (*P* = 0.0001) and the interaction effect (*P* < 0.0001) were all found to be significant. Statistically significant pairwise comparisons against FMDV A24 WT are represented in the figure by asterices and the color of the FMDV A24-P2/P3Deopt treatment group at the same time point. *p < 0.05, **p < 0.01, ***p < 0.001. Statistically significant pairwise comparisons between the 10^6^ and 10^7^ pfu FMDV A24-P2/P3Deopt treatment groups at each time point are represented with black daggers. ^†^*p* < 0.05, ^††^*p* < 0.01.

FMD vaccination prevents disease through the elicitation of a strong neutralizing antibody response in cattle. We observed that, following inoculation with the highest doses of A24-P2/P3Deopt, cattle in the 10^7^ pfu dose group developed neutralizing antibody titers at about the same rate as those animals administered the WT virus, with both groups reaching the protective threshold of 1.2 for cattle by 4 dpi (Fig. 3A). Meanwhile, those animals administered 10^6^ pfu of A24-P2/P3Deopt developed neutralizing antibody titers more slowly, reaching a protective threshold by 7 dpi and reaching a maximal titer somewhat lower than the other two treatment groups. A two-way ANOVA mixed effects model revealed highly significant effects for treatment group (*P* < 0.0001), time point (*P* = 0.0001) and treatment group x time point interaction (*P* < 0.0001). Post-hoc comparisons by timepoint revealed significant differences between WT and 10^6^ pfu administered cattle at 7 (*P* = 0.0225), 14 (*P* = 0.0009) and 21 dpi (*P* = 0.0068). A statistically significant difference was also detected between WT and 10^7^ pfu administered cattle at 14 dpi (*P* = 0.0359). Additionally, significant differences were detected between 10^6^ and 10^7^ pfu inoculated cattle, at 7 (*P* = 0.0192), 14 (*P* = 0.0213) and 21 dpi (*P* = 0.0025).

To determine the DIVA compliance of this vaccine, we next tested the sera at 0 and 21 dpi for antibodies against FMDV nonstructural (NS) proteins 3B_2_ and 3D^pol^ (PrioCheck®ELISA, Applied Biosystems) which had been mutated to serve as a DIVA marker in A24-P2/P3Deopt as in Uddowla et al. ^26^. We found that while 10^6^ pfu A24-P2/P3Deopt-administered animals remained free of any such antibodies, one animal from our maximum safety dose, 10^7^ pfu dose group developed these antibodies, leaving the DIVA status of this vaccine in doubt (Fig. 3B). Prior to inoculation, however, the virus was sequenced and the presence of the DIVA marker previously described^26^ was confirmed.

Our safety assessment of the FMDV A24-P2/P3Deopt strain reveals a virus that is safely administered even via IDL at high doses in cattle without risk of development of clinical disease, though its DIVA compliance needs further investigation.

### Efficacy study

**Fig. 4:**
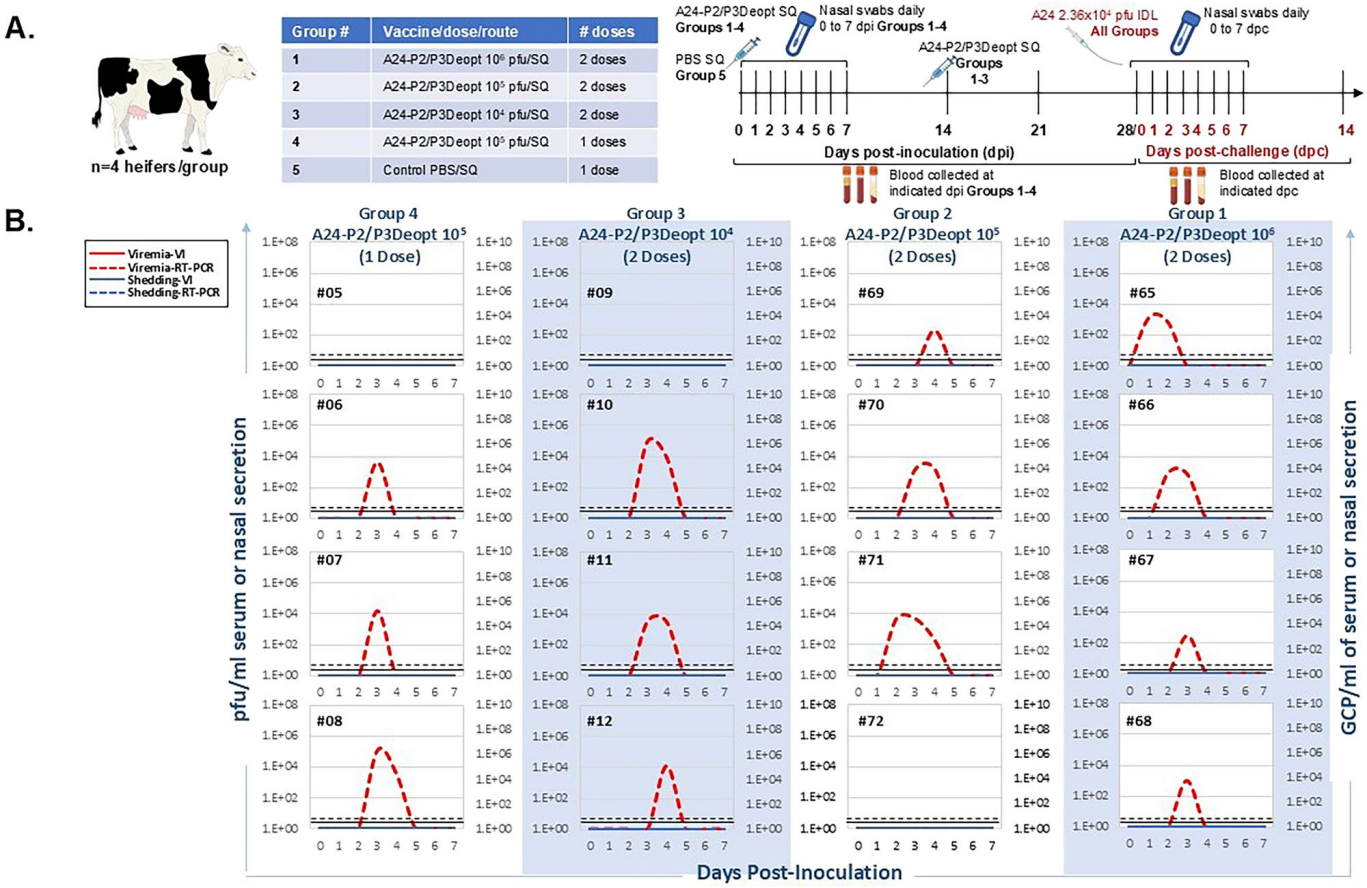
A24-P2/P3Deopt vaccine efficacy study design. **A** Six-month-old Holstein calves of approximately 450 lb were subcutaneously inoculated with an FMDV A24-P2/P3 codon deoptimized live attenuated virus either once (10^5^ pfu) or twice (10^4^, 10^5^ and 10^6^ pfu) prior to intradermolingual challenge with 10^4^ BID_50_ (2.36 × 10^4^ pfu) FMDV A24 wild type. Samples were collected at indicated time points after vaccine inoculation (dpi) and after challenge (dpc). **B** Viremia and viral shedding of live modified attenuated A24-P2/P3Deopt after vaccination (days post-inoculation). Viremia analyzed by virus isolation is expressed as pfu/ml of serum (solid red line). Viremia analyzed by real-time RT-qPCR is expressed as GCP/ml (dashed red line). Virus in nasal secretion analyzed by virus isolation is expressed as pfu/ml (solid blue line), while that analyzed by real-time RT-qPCR is expressed as GCP/ml (dashed blue line). Lower limits of detection are represented by a black dashed line for RT-qPCR and a solid black line for virus isolation. *n* = 4 cattle/time point/treatment group.

Having confirmed the safety of FMDV A24-P2/P3Deopt at high doses in cattle, we then moved on to assessing the effective dose range for preventing clinical disease upon homologous challenge. For this efficacy study, we utilized four groups of four Holstein heifers each and subcutaneously (SQ) administered FMDV A24-P2/P3Deopt as either a single dose (10^5^ pfu) or a two-dose series administered at 0 and 14 dpi (10^4^, 10^5^ and 10^6^ pfu). A fifth group of 4 heifers was subcutaneously administered PBS at 0 dpi (Fig. 4A). Animals in groups 1 through 4 were sampled daily through 7 dpi for blood and nasal secretions, then weekly thereafter for blood. Animals in group 5 were sampled at 0 dpi and 28 dpi/0 dpc to check for absence of virus prior to challenge. At 28 dpi, all animals were challenged with 2.36 x 10^4^ pfu (equivalent to 10^4^ BID_50_) of homologous FMDV A24Cruzeiro WT via the IDL route. Following challenge, cattle were clinically examined daily and again sampled daily for blood and nasal secretion up to 7 dpc, then weekly thereafter for blood.

To corroborate results obtained during safety studies, cattle were assessed for signs of clinical disease following primary SQ inoculation with FMDV A24-P2/P3Deopt. As expected, they were found to remain absent of fever, lymphopenia and the development of FMD vesicles (data not shown). Similar to the IDL-inoculated safety dose groups, shedding was absent via both virus isolation as well as RT-qPCR following inoculation (Fig. 4B). Additionally, viremia was absent by virus isolation, while variable amounts of circulating viral RNA (10^2^-10^7^ GCP/ml) were detectable—generally only for one day per animal—by RT-qPCR from timepoints ranging from 1-4 dpc.

**Fig. 5:**
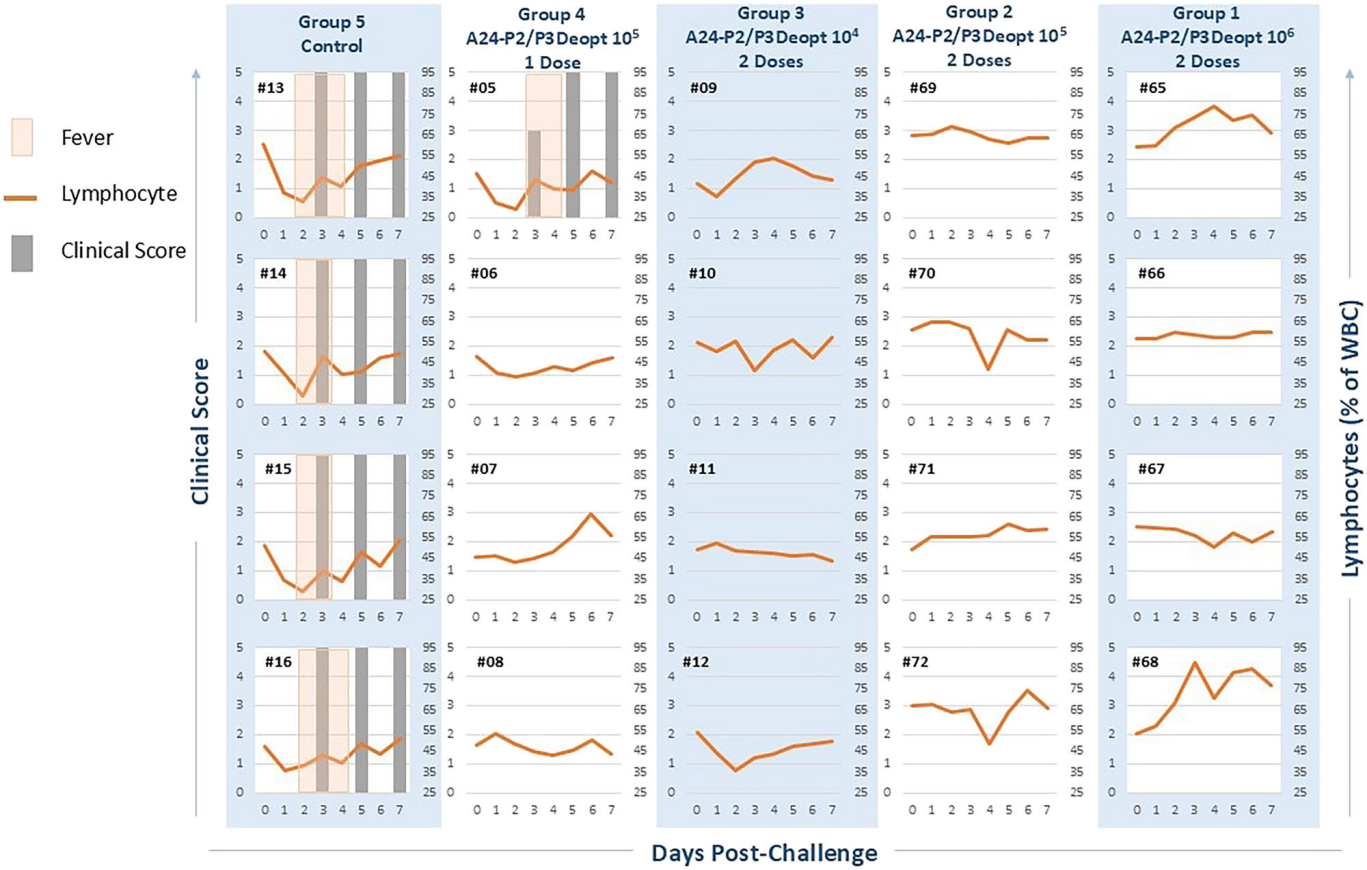
A24-P2/P3Deopt two dose vaccine regime protects cattle against homologous challenge. Six-month-old Holstein calves of approximately 450 lb were subcutaneously vaccinated with an FMDV A24-P2/P3 codon deoptimized live attenuated virus either once (10^5^ pfu) or twice (10^6^, 10^5^ or 10^4^ pfu) prior to intradermolingual challenge with 10^4^ BID_50_ (2.36 × 10^4^ pfu) FMDV A24 wild type. Cattle were assessed for clinical score (grey bars) on days 3, 5 and 7 post-challenge and rectal body temperature (periods of fever shown in tan shaded boxes), and percentage of lymphocytes on EDTA-treated blood (orange line) was assessed daily *n* = 4 cattle/time point/treatment group.

Following challenge at 28 dpi, all animals in the control group developed clinical FMD with elevated temperatures starting at 2 dpc and with maximum disease scores by 3 dpc (Fig. 5). On the contrary, all animals in the two-dose inoculation groups were found to be protected from the development of clinical FMD (Fig. 5). One heifer in the 10^5^ pfu single dose A24-P2/P3Deopt vaccinated group exhibited delayed fever (3-4 dpc) with a peak clinical score reached at 5 dpc, while the other 3 animals in this group were protected from disease after parental challenge.

**Fig. 6:**
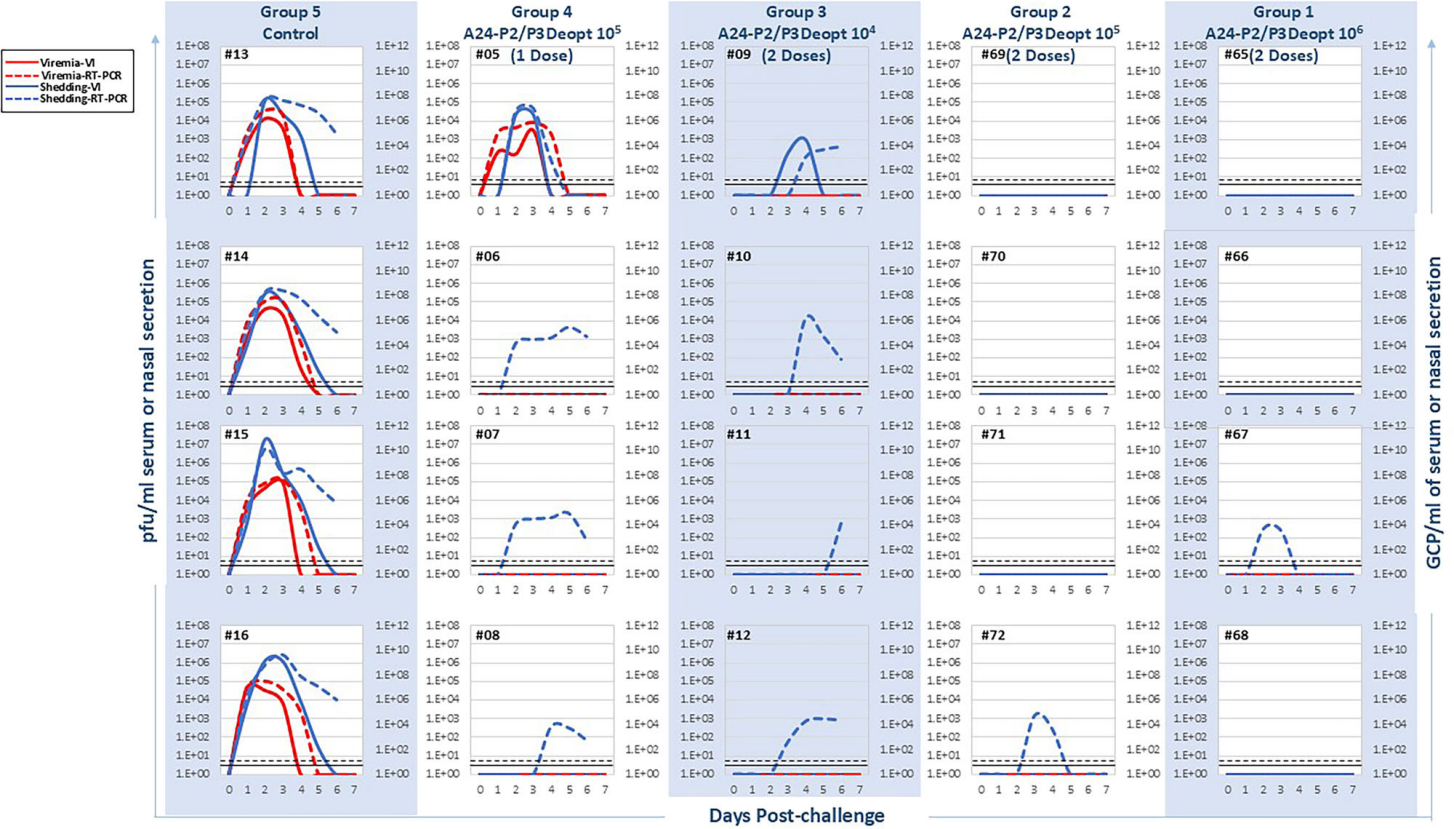
Viremia and virus shedding in A24-P2/P3Deopt efficacy study. Six-month-old Holstein heifers of approximately 450 lb were vaccinated with indicated doses of FMDV A24-P2/P3 codon deoptimized live attenuated virus and boosted at 14 dpi for 2-dose groups. At 28 dpi all animals were challenged with 10^4^ BID_50_ (2.36 × 10^4^ pfu) of homologous A24 WT virus intradermolingually (IDL). Serum and nasal secretion samples were collected daily for a week after challenge to assess viremia and virus shedding. Viremia analyzed by virus isolation is expressed as pfu/ml (solid red line). Viremia analyzed by real-time RT-qPCR is expressed as GCP/ml (dashed red line). Virus in nasal secretion analyzed by virus isolation is expressed as pfu/ml (solid blue line), while that analyzed by real-time RT-qPCR is expressed as GCP/ml (dashed blue line). Lower limits of detection are represented by a black dashed line for RT-qPCR and a solid black line for virus isolation. *n* = 4 cattle/time point/treatment group.

When cattle were assessed for viremia and shedding following challenge, a trend was observed in the inverse relationship between the dose (pfu) of vaccine received and the likelihood and amount of virus detected in both the blood and nasal secretions. (Fig. 6). Animals in the control group exhibited viremia and nasal shedding detected by both virus isolation and RT-qPCR starting as early as 1 day after challenge. Viremia peaked at titers of about 10^4^–10^5^ pfu/ml by virus isolation and 10^8^ GCP/ml by RT-qPCR, while shedding peaked at 10^5^–10^7^ pfu/ml by virus isolation and 10^7^–10^10^ GCP/ml by RT-qPCR. By contrast, none of the cattle inoculated with two doses of A24-P2/P3Deopt prior to challenge showed any viremia or RNemia. In parallel, shedding was observed in only 1 out of 4 heifers only by RT-qPCR at concentrations of about 10^4^ GCP/ml among the two groups vaccinated with the highest A24-P2/P3Deopt doses (two doses of 10^5^ or 10^6^ pfu). Amongst the two dose 10^4^ pfu efficacy group, 4/4 heifers displayed shedding by RT-qPCR with 1/4 also positive for shedding by virus isolation. Finally, among the single dose 10^5^ pfu dose group, the same animal that showed clinical FMD development also became viremic by 1 dpc by both RT-qPCR as well as virus isolation. Similarly, this animal began to shed by 2 dpc when assessed via virus isolation and RT-qPCR. All other heifers in this dose group were only positive for shedding by RT-qPCR and were negative for viremia.

**Fig. 7:**
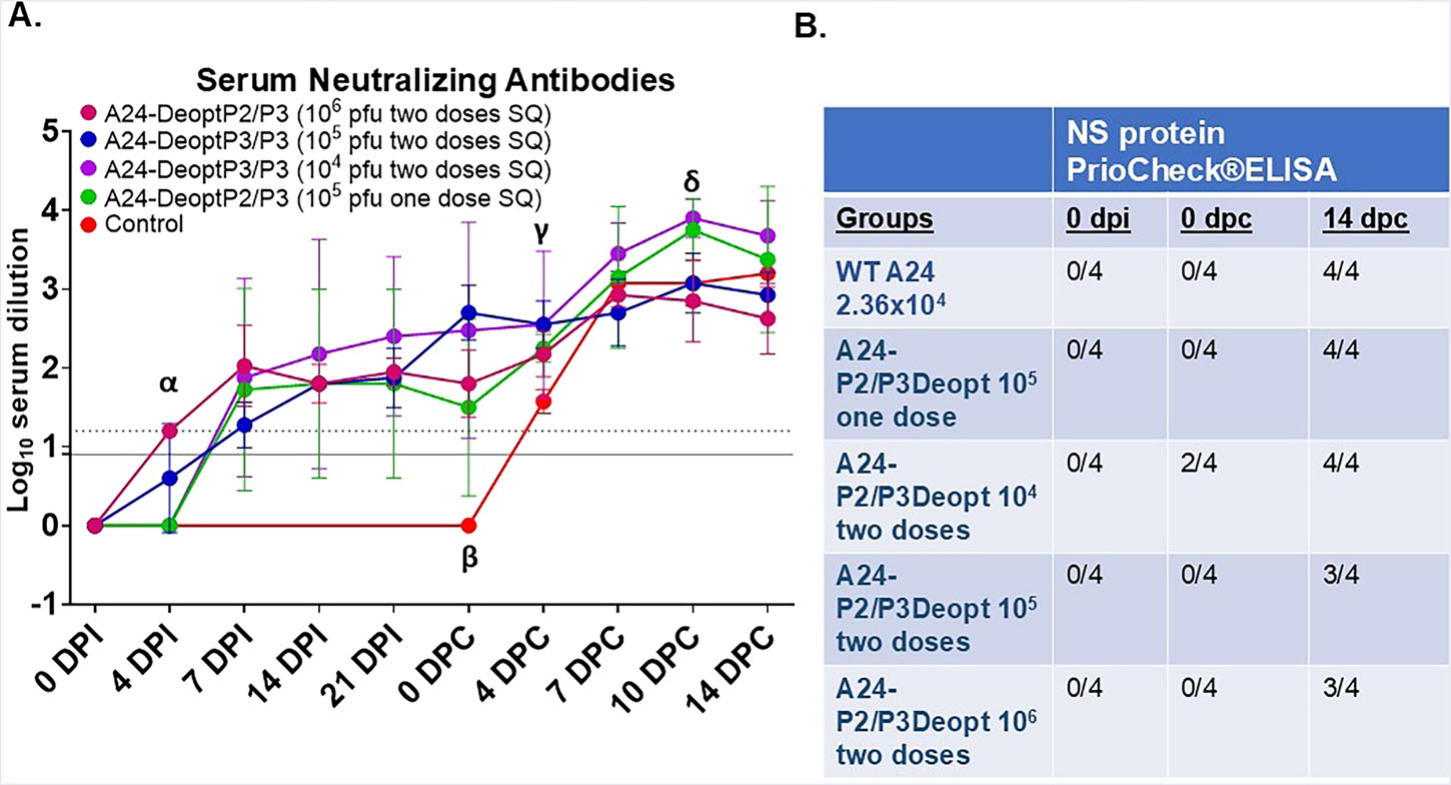
Adaptive immunity induced by A24-P2/P3Deopt vaccination. Six-month-old Holstein calves of approximately 450 lb were subcutaneously inoculated with an FMDV A24-P2/P3 codon deoptimized live attenuated virus either once (10^5^ pfu) or twice (10^6^, 10^5^ or 10^4^ pfu) prior to intradermolingual challenge with 10^4^ BID_50_ (2.36 × 10^4^ pfu) FMDV A24 wild type at 28 dpi. At indicated time points before and after challenge, serum samples were collected to test for **A** neutralizing antibodies (lower limit of detection represented by solid black line, while the protective titer is represented by a gray dashed line) or **B** antibodies against FMDV non-structural proteins (PrioCheck®ELISA). Number of positive samples out of the total number of animals per group. *n* = 4 cattle/time point/treatment group. Neutralizing antibody titers assessed by a mixed-effects two-way ANOVA by treatment group and time point, followed by pairwise comparisons at each time point by treatment group with Tukey’s multiple comparison correction. Treatment group (*P* < 0.0001), time point (*P* = 0.0016) and the interaction effect (*P* = 0.0047) were all found to be significant. **α**: Pairwise comparison at 4 dpi reveals that titers among cattle inoculated at 10^6^ pfu were significantly greater than those among both the one-dose 10^5^ pfu group and the two-dose 10^4^ pfu group (*P* < 0.0001 for both). **β:** Pairwise comparison at 0 dpc reveals that titers among cattle inoculated with two doses at 10^6^ pfu (*P* = 0.0135) and those inoculated with two doses at 10^5^ pfu (*P* = 0.0023) were significantly greater than titers among control animals. **γ**: Pairwise comparison at 4 dpc reveals that titers among cattle inoculated with two doses at 10^5^ pfu (*P* = 0.0156) and those inoculated with one dose at 10^5^ pfu (*P* = 0.0065) were significantly greater than titers among control animals. **δ**: Pairwise comparison at 10 dpc reveals that titers among cattle inoculated with two doses at 10^4^ pfu (*P* = 0.027) were significantly greater than titers among control animals.

Having established that a two-dose prime-boost vaccination strategy with FMDV A24-P2/P3Deopt can prevent clinical FMD at doses ranging from 10^4^–10^6^ when administered SQ, we next assessed the development of neutralizing antibody titers. We found that while all dose groups achieved a protective titer—as defined by a log_10_ serum dilution of ≥1.2 determined experimentally in our lab for other vaccine candidates^27^—on average by 7 dpi, the 10^6^ pfu dose group achieved that titer by 4 dpi (Fig. 7A). All vaccine dose groups maintained a protective titer leading up to challenge, at which point their serum neutralizing antibody titers increased again due to the boost effect of the challenge. A two-way ANOVA mixed effects model revealed significant effects for treatment (*P* < 0.0001), timepoint (*P* = 0.0016) and the interaction effect (*P* = 0.0047) between the two were all significant. Pre-challenge statistical comparisons of vaccinated groups against controls were not assessed, as blood samples were not collected from control cattle between 4 and 21 dpi during the pre-challenge period. At 4 dpi the neutralizing antibody titer of animals in the two-dose 10^6^ pfu treatment group was significantly higher than those from the single 10^5^ pfu dose and the two-dose 10^4^ pfu (*Ps* < 0.0001 for both) treatment groups. At 0 dpc, the neutralizing antibody titer of the cattle in the two-dose 10^6^ pfu treatment group (*P* = 0.0135) along with those in the two-dose 10^5^ pfu treatment group (*P* = 0.0023), were found to be significantly higher than the control animals, whose titer remained at 0. At 4 dpc the neutralizing antibody titer of the cattle in the two-dose 10^5^ pfu treatment group (*P* = 0.0156) along with those in the one-dose 10^5^ pfu treatment group (*P* = 0.0065) were found to be significantly higher than the control animals, and at 10 dpc, neutralizing antibody titers of the cattle from the two-dose 10^4^ pfu treatment group were found to be significantly higher (*P* = 0.027) than that of the control group.

As with the safety study, we again looked to assay for the presence of anti-NSP antibodies in vaccinated cattle to determine the DIVA compliance of this LAV candidate (Fig. 7B). Curiously, we found that all animals remained anti-NSP antibody negative following vaccination except for two heifers from the two-dose A24-P2/P3Deopt 10^4^ pfu dose group. By 14 dpc, all animals from the control, two-dose 10^4^ and one-dose 10^5^ A24-P2/P3Deopt dose groups were anti-NSP antibody positive, while 3/4 heifers were positive from each of the remaining two-dose 10^5^ and 10^6^ dose groups. The results at 0 dpc again cast doubt on the DIVA compliance of this vaccine candidate as formulated and with the current available tests, and indicate that further tweaking of the sequence may be necessary for true DIVA compliance.

**Fig. 8:**
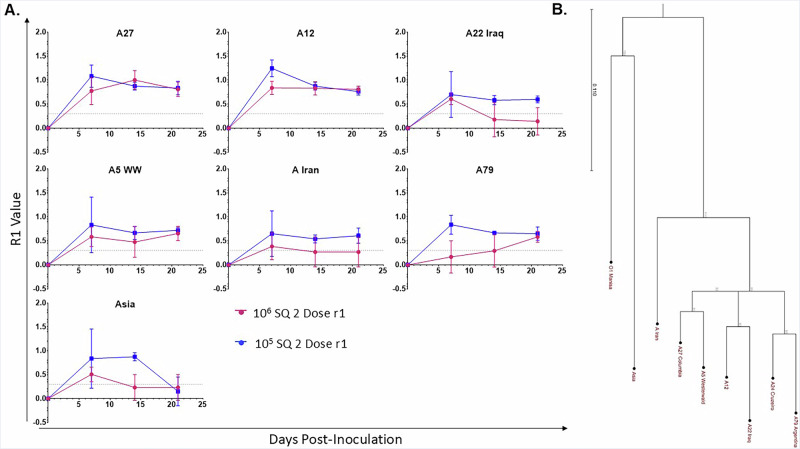
Vaccination with A24-P2P3Deopt elicits a broadly-neutralizing antibody response in cattle. Six-month-old Holstein calves of approximately 450 lb were subcutaneously inoculated with an FMDV A24-P2/P3 codon deoptimized live attenuated virus twice at doses of 10^6^ or 10^5^ pfu. **A** Serum samples were collected at specified time points after inoculation to measure neutralizing antibodies against various serotype A strains of FMDV, as well as Asia1 and O1Manisa (data not shown). The results are presented as r1 values. The dotted line at r1 = 0.3 represents the ratio generally considered to be protective. **B** Phylogenetic analysis of the P1 region sequence identity of the tested strains compared to A24Cruzeiro. *n* = 4 cattle/time point/treatment group.

Finally, we were interested in assessing the broadly neutralizing capacity of the antibodies elicited by animals inoculated with LAV candidate. To that end, we tested the sera of two-dose 10^5^ and 10^6^ pfu A24-P2/P3Deopt inoculated cattle from 0, 7, 14 and 21 dpi against six other serotype A strains, and two different serotype strains, Asia1 and O1Manisa (Fig. 8A) and used the resulting titers to calculate r1 value (defined as the quotient of the titer against the heterologous strain over the titer against the homologous strain) for each strain. In general, sera from the two-dose 10^5^ pfu dose group consistently performed as well or better than samples from the two-dose 10^6^ pfu dose group against a variety of serotype A strains, both in the aggregate and over time. Titers continued to rise or remain steady up to 21 dpi in all A strains except A12, where a slight decline in average titer was observed at 21 dpi, though the titer and r1 value continued to exceed the threshold for protection. In contrast, the sera from the two-dose 10^6^ pfu A24-P2/P3Deopt inoculated cattle showed a decline in neutralizing capacity by 14 dpi (A22 Iraq and A Iran) or by 21 dpi (A27) in three of six A strains tested. Additionally, when tested against A79, sera from the two-dose 10^6^ pfu A24-P2/P3Deopt inoculated animals took longer to achieve an average protective r1 value of 0.3 (14 dpi vs 7 dpi) than the two-dose 10^5^ pfu A24-P2/P3Deopt dose group. While no titer was obtained against O1Manisa at any timepoint (data not shown), the sera of the two-dose 10^5^ pfu dose group reached a neutralizing titer as well as an r1 value greater than 0.3 against serotype Asia1 by 7 dpi, though the titer returned to 0 by 21 dpi. Phylogenetic analysis of VP1 (Fig. 8B, S1 Fig) from the strains used for the cross-protection neutralization assay reveals low correlation with the r1 values obtained by this assay. However, while r1 values remained strong over the various timepoints tested (7, 14 and 21 dpi) for all serotype A strains, particularly among the A24-P2/P3Deopt 10^5^ pfu dose group, r1 values fell to just above zero by 21 dpi for the more distantly related Asia1 strain.

## Discussion

Our work demonstrates that the A24-P2/P3Deopt strain is a highly effective live attenuated vaccine candidate with a broad safety margin and ability to elicit a strong, cross-protective neutralizing antibody response when administered in cattle, improving current vaccine strategies. Specifically, we have demonstrated that A24-P2/P3Deopt is safe at IDL doses as high as 10^7^ pfu and effective at a prime-boost dose of 10^4^ pfu, establishing a safety margin in cattle of at least three logs.

LAVs are generally understood to be the most successful type of vaccine, not only because they typically induce stronger protection, but because they also bring other advantageous qualities that make them easier to deploy than other vaccine types commonly applied to viral illnesses. For example, all commercial LAVs—with the exception of the oral polio vaccine—are lyophilized, a process of freeze-drying that results in significantly improved stability, sterility of the end-product and reduced reliance on cold chain^28^. This reduced reliance on cold chain also significantly reduces the cost of producing these vaccines and improves their distribution to remote and low-resource areas for field application. In addition to cost savings from reduced cold chain reliance, LAVs also require less viral material per dose. For example, the commercially available FMD vaccine formulation is administered at 10 µg/dose, an amount equivalent to about 2 x 10^8^ pfu (using FMDV A12 for demonstrative purposes, which has a molar mass of 8.6 x 10^6^ g/mol and a viral particle-to-pfu ratio of 5000 VP/pfu^21^). By comparison, while the viral particle-to-pfu ratio of the present A24-P2/P3Deopt LAV is 27,000 VP/pfu, at the lowest effective dose schedule investigated—two doses at 1 x 10^4^ pfu each—the viral material administered in a commercially inactivated vaccine is still 3 logs higher than for A24-P2/P3Deopt. Production cost of codon deoptimized FMD vaccines could also be further reduced if the CD strain were delisted on the United States Select Agent Program regulations and able to be grown outside of BSL-3 facilities, as has happened for other FMD attenuated vaccine strains such as FMDV A24 Leaderless^29^.

Another benefit of LAVs is their ability to confer early, long-lasting and broad-spectrum immunity by recruiting both humoral and cellular arms of the immune system. In the past, while several attenuated strains tested in cattle were not capable of inducing protective levels of immunity, swine inoculated with FMDV SAP mutant were protected against WT homologous challenge as early as 2 days after inoculation^30^. In the present study, animals inoculated with the highest dose in the efficacy study (10^6^ pfu), showed systemic protective antibody titer by 4 dpi, at least as rapidly as the adjuvanted inactivated FMD vaccine formulation^5^, suggesting the present LAV vaccine could be an effective substitute and may be protective even at this early time point, though a challenge study should be conducted to confirm this hypothesis.

While the precise mechanisms of attenuation conferred by CD or CPD are not well-understood, particularly in the context of FMDV, many well-supported hypotheses exist. First, CPD has been shown to increase CpG and UpA dinucleotide frequencies in the resulting viruses (as reviewed in ref. ^31^). These dinucleotide pairs are far less frequent in mammalian hosts, and many innate immune sensing molecules exist that target them, triggering an antiviral response that slows virus growth and spread among cells. Additionally, the increase in these uncommon dinucleotide pairs is attended by increasing frequency of “near-stop” codons, which are more prone to random mutation into stop codons, leading to evolutionary dead-ends. It has been long since established that tRNA concentrations for uncommon codons tend to be lower, which may contribute to a reduced rate of translation^32^. CPD is also understood to have effects on RNA secondary structure folding, which can result in changes in translation initiation and stalling and can further lead to misfolding of the amino acid chain (as reviewed in ref. ^33^). All these putative mechanisms of attenuation result in viruses that are slow growing, which presents a drawback for large-scale production. Given the slower growth rate, CPD viruses are more time and resource intensive than WT viruses. However, the cell lines used in virus propagation could be modified to make them more permissive to replication of these CPD viruses with reduced fitness. For instance, the host factor variously known as zinc-finger antiviral protein (ZAP) and poly(ADP-ribose) polymerase 13 (PARP13) among others, is a viral nucleic acid sensor and a component of the innate immune system. ZAP targets and binds CpG dinucleotides in RNA and targets them for destruction^34^. In the future, cell lines with knock-outs or RNA binding domain mutations in antiviral proteins such as ZAP could be developed to improve CPD vaccine strain production. On the other hand, CD viruses showed a reduced translation efficiency because rare tRNAs are limiting, causing the ribosome to stall or pause, ultimately reducing protein yield and therefore influencing viral fitness^18,19,35^. CD also has an effect on viral protein folding kinetics due to slowed translation^36^. Therefore, both methods of deoptimization ultimately cause viral attenuation.

In the present study, we observed a lack of detectable circulating virus by virus isolation and only low levels by PCR in some cattle, consistent with our previous findings in swine^23^. It was proposed in the previous swine study that the lack of detectable circulating virus following vaccination likely contributed to the suboptimal humoral immune response. However, the robust humoral immune response observed in vaccinated cattle despite the recapitulation of the viremia results seen earlier in swine suggests that the cause of the reduced humoral immunogenicity of this virus in swine is likely more complex and multifactorial than a lack of circulation and may instead be related to swine immunology or the tropism of the A24-P2/P3Deopt strain. Immunological divergence has been observed between cattle and swine. For example, while IgA^+^ B cells—a critical contributor to mucosal immunity—are found circulating only in small numbers in swine^37^, an analysis of the frequency of expressed sequence tags in GenBank suggests that IgA^+^ B cells may account for as many as 15% of the circulating B cell population in cattle (unpublished data). A robust circulating double-positive CD4^+^CD8^+^ T cell population has also been observed in suids and seems to play a role in immune response to certain pathogens, such as African swine fever virus^38–40^ and Japanese encephalitis virus^41^, while this population circulates at a much lower frequency in cattle, though may still be relevant to pathogenesis in certain diseases^42^. Additionally, the frequency of regulatory T cells (Treg) in cattle has been observed at high levels relative to many other mammalian species^43^, while this cell population represents a smaller subset of porcine CD4^+^ T cells^44,45^. Tregs are well known to play a role in virus-host détente and persistence^46^, and it has been proposed that they may be important to virus ecology in cattle in the context of FMDV^47^.

Many differences in FMDV immunity specifically have also been observed over the years. For example, while a strong neutralizing antibody titer reliably correlates with a protective immune response in cattle, this is not the case in swine^48–50^. In cattle immunized with the adenoviral vectored FMDV O1Campos vaccine, the proportion of CD4^+^ and CD8^+^ cells expressing IFNγ upon ex vivo restimulation significantly increased following a homologous challenge against which they were protected^51^. Similarly, PBMCs isolated from cattle inoculated with the inactivated FMD vaccine either with or without Montanide adjuvant produced significant quantities of IFNγ at 4- and 7-days post-vaccination^5^. While the present LAV platform has not demonstrated protective efficacy—even serotype specific—in swine, it is possible that this could be achieved through formulation with adjuvants or using alternative routes of vaccination. While our group found that a preparation of Asia1 Shamir CPD formulated with Montanide did not result in protective efficacy when administered to swine (data not published), novel adjuvants could be assayed. Analogously, novel routes of vaccination that increase potency and safety might be considered, such as microneedle inoculation^52^.

In addition to demonstrating a strong humoral immune response and excellent efficacy against the parental A24Cruzeiro WT strain, sera from the two-dose 10^6^ and 10^5^ pfu A24-P2/P3Deopt vaccinated groups demonstrated broad neutralization capacity both within the A serotype and even across serotypes against Asia1. This is consistent with what has been shown historically with a wide variety of live attenuated vaccines^53,54^, underscoring the vaccine’s potential for application in ever-evolving outbreak situations. For many of the other strains assayed by serum microneutralization, the amino acid sequence identity of VP1—particularly in hypervariable regions such as the G-H loop and AA43-49, both regions known to interact with integrin during receptor binding and internalization—did not necessarily concord strongly with the r1 value of the assay. This suggests that other immunodominant epitopes in the capsid may be responsible for driving the broadly neutralizing response observed early after vaccination or that successful neutralization may be more dependent on microhomology within these hypervariable regions, such as the RGD sequence in the G-H loop. When considering O1Manisa, it is possible that the incorporation of charged and polar amino acids well-suited to electrostatic interactions in the G-H loop contributed to the lack of cross-reactivity of the serum antibodies. In addition to the broadly neutralizing antibody response elicited by this LAV vaccine, the vector from which the A24-P2/P3Deopt virus was derived was engineered with restriction sites flanking the P1 capsid domain, allowing for easy sequence swapping to quickly derive additional vaccine candidates against other serotypes and strains^26^. As a result, this platform has the potential to be rapidly adapted to meet local needs in outbreak situations.

Currently, the predominant trend in FMD vaccination strategy is to utilize vaccines incorporating purification steps in the production chain or incorporating DIVA markers. Unfortunately, it remains unclear why the DIVA marker included in A24-P2/P3Deopt^26^ is not fully effective. Given the error-prone RNA polymerase of this virus, however, it is conceivable that random mutations over serial passage resulted in some reversion to parental sequence. In their work on a CPD RSV vaccine strain, MIN AL, Levy et al. found significant accumulation of mutations over serial passage, though most were missense mutations that did not result in reversion to virulence^17^. However, in their work with MIN AL, they found that reintroduction of a few point mutations was sufficient to reverse virus temperature sensitivity and return growth curves to the levels seen in WT RSV. Conversely, Yeh et al. ^55^. was able to introduce significant genetic stability over many passages to the Sabin2 live attenuated poliovirus—vaccines notorious for rapid genetic evolution and reversion to virulence—by introducing a small number of mutations in 3 regions of the genome, including mutations in the 5’ UTR to stabilize a temperature sensitivity mutation and move a cis-acting replication element (*cre*), several synonymous mutations in 2C to disrupt the secondary structure of a *cre* site and single amino acid substitutions in 3D. For the present FMD vaccine candidate to be attractive for licensing, the issue around DIVA marker stability must be further investigated. Reversion to virulence is always a concern with LAVs, and while recombination events could be initiated in the UTRs or non-deoptimized 3D region of A24-P2/P3Deopt, we have previously shown that the risk of such recombination events is low^56^. Additionally, such recombination events would require the presence of WT virus. Mutations to the 3D^pol^ gene that increase replication fidelity, such as replacement of W237 with phenylalanine, have resulted in mutants with similar in vitro growth kinetics to WT virus, while achieving a more attenuated in vivo phenotype^57^. Such mutations could also be incorporated into future infectious clones, although it has been observed that the non-deoptimized region (conserved because of a proposed RNA structure at this site^58^) of the 3D^pol^ in A24-P2/P3Deopt served as a recombination initiation site when WT virus was present in vitro^56^. While genetic stability over many passages should be assessed, both in vitro and in vivo, recombination events such as this in the field with WT virus would not result in more virulent strains of virus than the currently circulating WT.

The present study demonstrates that our A24-P2/P3Deopt vaccine candidate shows strong promise in cattle as a LAV vaccine. Not only have we established a minimum 3-log safety margin for this strain and a lack of shedding, A24-P2/P3Deopt induced full protection and elicited broad cross-neutralizing antibody protection. Given these factors and the engineering of this live attenuated virus, this platform could provide an agile tool for outbreak prevention and control.

## Methods

### Virus and cells

Porcine kidney cells (LFPK) were obtained from the Foreign Animal Disease Diagnostic Laboratory (FADDL) of the Animal and Plant Health Inspection Service (APHIS) at the Plum Island Animal Disease Center (PIADC). A porcine kidney cell line overexpressing specific integrin receptors (LFPK-αVβ6) was developed in-house as previously described^59^. BHK-21 (Baby hamster kidney cells, strain 21, clone 13, ATCC CL10) were purchased from the American Type Culture Collection (ATCC, Manassas, VA). All cells were maintained as previously reported^60^. Stock of FMDV A24 WT was generated from the full-length serotype A24Cruzeiro infectious clone (pA24-WT) and amplified in BHK-21 cells as previously reported^61^. Stock of FMDV A24-P2/P3Deopt was generated as described by Diaz-San Segundo et al. ^23^ and amplified in LFPK-αVβ6 as previously reported by the same group. Specifically, the P2 and P3 regions of the A24 WT genome were codon deoptimized and antigenic markers at 3B and 3D^pol^ were abolished through a RQKP_9-12_ → PVKV substitution in 3B_2_, along with H_27_Y and N_31_R in 3D^pol^ ^26^ to serve as a DIVA marker.

### Animal studies

All animal experiments were performed under agricultural biosafety level 3 at Plum Island Animal Disease Center (PIADC) and were approved by the PIADC Institutional Animal Care and Use Committee of the U.S. Department of Agriculture and the U.S. Department of Homeland Security (USDA/APHIS/AC Certificate number: 21-F-0001; Protocol #: 244.00-23-R). Furthermore, all experiments were completed in compliance with: the Animal Welfare Act (AWA) (2011); Guide for the Care and Use of Laboratory Animals; the 2002 Public Health Service Policy for the Humane Care and Use of Laboratory Animals; U.S. Government Principles for Utilization and Care of Vertebrate Animals Used in Testing, Research and Training (IRAC, 1985); as well as specific animal protocols reviewed and approved by the Institutional Animal Care and Use Committee (IACUC) of the Plum Island Animal Disease Center (USDA/APHIS/AC Certificate number: 21-F-0001; Protocol 244.01-19-R).

A total of 32 Holstein heifers (*n* = 4 heifers/treatment group) of about 450 lbs each were used for the current study. In a preliminary safety study, 2 groups were intradermolingually inoculated with either 10^6^ or 10^7^ pfu of A24-P2/P3Deopt and a control group was inoculated with 2.36 × 10^4^ pfu [equivalent to 10^4^ 50% bovine infectious dose (BID_50_)] wild-type A24Cruzeiro (A24 WT) virus. Heifers were then followed for up to 21 days post inoculation (dpi). In a follow-up efficacy study, four efficacy groups were subcutaneously inoculated/vaccinated either only once with 10^5^ pfu A24-P2/P3Deopt or twice at 0 and 14 dpi with 10^6^, 10^5^ or 10^4^ pfu, plus a control group inoculated with PBS. At 28 dpi, all cattle were challenged with 2.36 × 10^4^ pfu (equivalent to 10^4^ BID_50_) A24 WT IDL and followed out to 14 days post-challenge (dpc).

Clinical evolution in both the safety and efficacy studies was followed on a daily basis for 7 days after inoculation and after challenge, with temperature recorded every day and clinical score assessment at 0, 3, 5 and 7 dpi/dpc. Clinical score is calculated as the sum of the number of feet and the snout that present with vesicular lesions, with a maximal score of 5. Nasal swabs were collected daily from 0-9 dpi/dpc. The blood collection schedule is detailed below.

For intradermolingual inoculation as well as clinical evaluations of the snout and feet occurring on 0, 3, 5 and 7 dpi/dpc, animals were sedated with IM xylazine at 0.66 mg/kg and reversed by IV tolazoline at 2 mg/kg. At the conclusion of the study, animals were humanely euthanized by IV pentobarbital sodium overdose at 85 mg/kg followed by exsanguination.

### Blood processing

Blood samples were collected daily from 0-9 dpi/dpc, then weekly at 14 and 21 dpi/dpc in serum or EDTA Vacutainers (BD, Franklin Lakes, NJ). Serum tubes were allowed to coagulate for at least 30 min and centrifuged at 2100 × *g* for 10 min at RT before aliquots were collected. Half the aliquots were heat-inactivated at 56 °C for 30 min and all samples stored at −70 °C until analysis. Blood from EDTA tubes was analyzed on a Hemavet (Drew Scientific, Miami Lakes, FL).

### Anti-NSP ELISA

In order to test the DIVA compliance of this vaccine, serum samples from 0 dpi and 21 dpi in the safety study, and 0 dpi, 0 dpc and 14 dpc in the efficacy study were assayed for presence of anti-nonstructural proteins (NSP) antibodies targeted to viral genes 3B and 3D^pol^ by PrioCHECK™ competitive ELISA (Applied Biosystems, Waltham, MA) according to manufacturer’s instructions.

### Serum neutralization assay

Serum neutralization assays were performed on heat-inactivated cattle sera samples by end-point titration according to the Kärber method^62^ on BHK-21 cells. Antibody titers were expressed as the log_10_ value of the reciprocal of the dilution that neutralized 100 TCID_50_ WT A24Cruzeiro in 50% of the wells.

### Detection of virus in sera and nasal swabs

Cattle sera and nasal secretion were examined for the presence of virus by plaque assays on BHK-21 cells for post-inoculation samples and on LFPK-αVβ6 cells for post-challenge samples to account for differences in virus strain susceptibility of the two cell lines. Virus titers were expressed as log_10_ pfu/ml of serum or nasal swab secretions. The minimal detection level for this assay is 5 pfu/ml. In addition, FMDV RNA was detected by real-time quantitative PCR (RT-qPCR) as previously described^63^. Cycle threshold (Ct) values were converted to RNA copies per milliliter using the equation derived from analysis of serial 10-fold dilutions of in vitro synthesized FMDV RNA of known concentration and expressed as the genome copy number per ml of serum or nasal swab^64^.

### Phylogenetic analysis

FMDV sequences were acquired from National Center for Biotechnology Information (NCBI), A12 (GenBank #M10975), A24Cruzeiro (#AY593768), O1 Manisa (#AY593823), A Iran (# MZ493234), A22 Iraq (#AY593764.1), A5 Westerwald (# AY593781), Asia (# NC_004915), A27 Columbia (# AY593771). Sequences were aligned using ClustalW and trees were constructed using the Maximum Likelihood Phylogeny programs in CLC Genomics Workbench v. Version 23.0.2. Sequence identity was established using the Bioinformatics.org Sequence Manipulation Suite (Last updated October 1^st^, 2023).

### Statistics

Data handling, analysis and graphic representations were performed using Prism 10.6 (GraphPad Software, San Diego, CA) or Microsoft Excel (Microsoft, Redmond, WA). Neutralizing antibody titers were assessed by a mixed-effects two-way ANOVA by treatment group and timepoint followed by pairwise comparisons with Tukey’s multiple comparisons correction.

## Supplementary information

Supplement.

## Data Availability

Data is provided within the manuscript or supplementary information files. Further inquiries can be directed to the corresponding authors.
