# Supplementary material for: Live-attenuated foot-and-mouth disease virus vaccine engineered by codon deoptimization induces a strong protective immune response in cattle
Source: NPJ Vaccines. 2026 Jan 24;11:48. doi: 10.1038/s41541-025-01368-7 (PMC12894751; doi:10.1038/s41541-025-01368-7)
Supplement: Supplementary file 1 — Supplement. [file 41541_2025_1368_MOESM1_ESM.pdf]

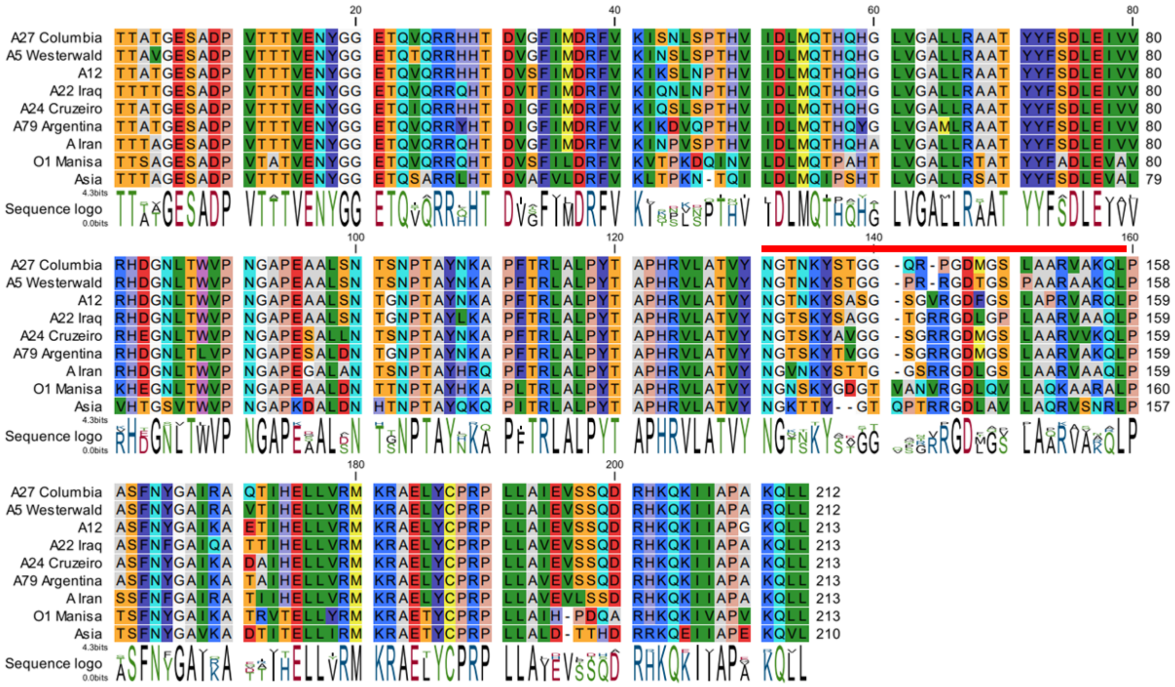

	A27Col	A5WW	A12	A22Iraq	A Iran	O1M	Asia1	A79
G-H Loop	72	64	64	72	64	38.5	36	92.9
Full Length	92	90.6	90.1	88.7	84.5	71.5	66.7	92.5

S1 Fig. Amino acid sequence alignment between VP1 region of cross-neutralization strains. Amino acid sequence alignment performed on the nine FMDV strains used for serum neutralization assay at 0, 7, 14 and 21 dpi, including A24 Cruzeiro. G-H loop sequence is identified by a red bar and the percentage sequence identity with A24 Cruzeiro is provided in the included table.
